# Prokaryotic Expression and Serodiagnostic Potential of Glyceraldehyde-3-Phosphate Dehydrogenase and Thioredoxin Peroxidase from *Baylisascaris schroederi*

**DOI:** 10.3390/genes8110293

**Published:** 2017-10-25

**Authors:** Yu Li, Ying Sun, Xiaobin Gu, Yue Xie, Weiming Lai, Bo Jing, Xuerong Peng, Guangyou Yang

**Affiliations:** 1 Department of Parasitology, College of Veterinary Medicine, Sichuan Agricultural University, No.211, Huimin Road, Chengdu 611130, China; amberlee531@hotmail.com (Y.L.); Chengguo_92@hotmail.com (Y.S.); guxiaobin198225@126.com (X.G.); zhandegaokandey123@163.com (Y.X.); aliciaheml@hotmail.com (W.L.); jingbooo@163.com (B.J.); 2 Department of Chemistry, College of Life and Basic Science, Sichuan Agricultural University, Chengdu 611130, China; chunyanli@163.com

**Keywords:** giant panda, glyceraldehyde-3-phosphate dehydrogenase, Thioredoxin peroxidase, immunofluorescence, indirect ELISA

## Abstract

*Baylisascaris schroederi*, a roundworm parasite of giant pandas, badly affects the health of its hosts. Diagnosis of this disease currently depends mainly on sedimentation floatation and Polymerase Chain Reaction (PCR) methods to detect the eggs. However, neither of these methods is suitable for diagnosis of early-stage panda baylisascariasis and no information on early diagnosis of this disease is available so far. Therefore, to develop an effective serologic diagnostic method, this study produced recombinant glyceraldehyde-3-phosphate dehydrogenase (GAPDH) and thioredoxin peroxidase (Tpx) proteins from *B. schroederi* using a prokaryotic expression system. We determined the immunological characteristics of these proteins and their location in the parasite. Indirect enzyme-linked immunosorbent assays (ELISAs) were established to detect *B. schroederi* infection in giant pandas based on GAPDH and Tpx respectively. The open reading frame of the *GAPDH* gene (1083 bp) encoded a 39 kDa protein, while the predicted molecular weight of Tpx (588 bp) was 21.6 kDa. Western-blotting analysis revealed that both recombinant proteins could be recognized with positive serum of pandas infected with *B. schroederi*. Immunohistochemical staining showed that the endogenous GAPDH of *B. schroederi* was widely distributed in the worm while Tpx was mainly localized in the muscle, eggs, gut wall, uterus wall and hypodermis. Serological tests showed that the GAPDH-based indirect ELISA had a sensitivity of 95.83% and specificity of 100%, while the test using Tpx as the antigen had sensitivity of 75% and specificity of 91.7%. Thus, *B. schroederi* Tpx is unsuitable as a diagnostic antigen for baylisascariasis, but *B. schroederi* GAPDH is a good candidate diagnostic antigen for *B. schroederi* in pandas.

## 1. Introduction

The giant panda (*Ailuropoda melanoleuca*) is well known as a conservation flagship species and a “living fossil”. There are 471 captive giant pandas distributed in 84 institutions in 18 countries according to pedigree data in 2016 (unpublished). *Baylisascaris schroederi* is a common roundworm parasite of the intestine of giant pandas [1]. This parasite may also occasionally be detected in the mouth, stomach, larynx and trachea, as a result of migration through the definitive host [2,3]. It is reported that the positive rate of *B. schroederi* eggs in captive panda feces is about 25.71% [4]. Hosts infected with this worm may have clinical symptoms such as malnutrition, fasting, emesis, diarrhea, emaciation, cough, cachexia among others [5]. In addition, adult roundworms may migrate to pancreatic ducts and biliary ducts, and the host may suffer from pancreatitis and pneumonia because of the extensive damage of organs. A large quantity of parasites may also cause intestinal obstruction of giant pandas, and even death [6,7]. The diagnosis of this disease mainly depends on sedimentation floatation and Polymerase Chain Reaction (PCR) methods to detect the eggs [8,9,10]. However, it is a significant challenge to carry out a diagnosis at an early stage of infection using these methods.

Thioredoxin peroxidase (Tpx) is widely present in eukaryotes and prokaryotes [11]. The main function of this protein is mopping up superfluous reactive oxygen in tissues [12,13,14]. Several Tpx-based enzyme-linked immunosorbent assay (ELISA) methods have been developed and the results demonstrate that Tpx is a candidate diagnostic antigen for some parasitic diseases [15,16,17]. Glyceraldehyde-3-phosphate dehydrogenase (GAPDH or G3PDH) is a multifunctional enzyme in the metabolism of parasites and often used in quantitative PCR as a housekeeping gene [18,19]; the function of this protein depends on its subcellular location [20]. It is known that the excreted/secreted products of parasites can induce humoral responses and produce circulating antibodies in their definitive hosts. Several studies have shown that GAPDH presented in excretory/secretory products of *Schistosoma japonicum* and can stimulate a short-lived antibody response in hosts [21,22], suggesting that GAPDH may be useful for serological diagnosis of parasitic diseases [23].

Until now, there have been no reports on GAPDH or Tpx of *B. schroederi.* Thus, the goal of this study was to express these proteins using a prokaryotic expression system and further explore their immunogenicity and localization in *B. schroederi*. Meanwhile, the indirect ELISAs based on either GAPDH or Tpx was also established for serodiagnosis of baylisascariasis.

## 2. Materials and Methods

### 2.1. Parasites and Animals

Adult *B. schroederi* and infective embryonated eggs derived from the feces of giant pandas were stored and provided by the Department of Parasitology, College of Veterinary Medicine, Sichuan Agricultural University.

Four male New Zealand White rabbits (1.2–2.0 kg) were obtained from the Laboratory Animal Center of Sichuan Agricultural University and 20 female ICR mice (specific-pathogen-free grade) of 7-weeks-old were purchased from Chengdu Dashuo Animal Experimental Center. All animals were housed in a barrier environment in sterile cages and provided with pelleted food and sterilized water *ad libitum*. They were acclimated to these conditions for 1 week before experiments. 

### 2.2. Serum and Secondary Antibody

Twenty-four positive serum samples were isolated from giant pandas naturally infected with *B. schroederi* in Wolong Giant Panda Protection and Research Center. These infected pandas were monitored as vomiting or excreting *B. schroederi* over a long period, and *B. schroederi* eggs were detected in their feces by traditional sedimentation floatation assay [24]. Thirty-six negative serum samples were collected from *B. schroederi*-free giant pandas in Chengdu Research Base of Giant Panda Breeding. No *B. schroederi* worms or eggs were found in these negative pandas for 3–4 months. All sera were stored at −20 °C before use. Specific horseradish peroxidase (HRP)-labeled rabbit anti-panda secondary antibody was prepared by Chengdu Zheng Neng Biotechnology Co., Ltd., Chengdu, China.

### 2.3. Ethics Statement

Animals were handled strictly according to the animal protection law of the People’s Republic of China (released on 18 September 2009) and the National Standards for Laboratory Animals in China (executed on 1 May 2002). This study was reviewed and approved by the Animal Ethics Committee of Sichuan Agricultural University (China) (Approval No. 2013-028). All the methods were carried out in accordance with all relevant guidelines and regulations.

### 2.4. RNA Extraction and Amplification of Baylisascaris schroederi Thioredoxin Peroxidaseand Glyceraldehyde-3-Phosphate Dehydrogenase 

Total RNA was extracted from *B. schroederi* adults isolated from giant pandas and reverse transcribed into cDNA according to the recommendation of the manufacturer (Fermentas, Shenzhen, China) and stored at −70 °C. Subsequently, the complementary DNA (cDNA) was used as a template to amplify the *Baylisascaris schroederi*-*Tpx* (*Bs-Tpx*) gene and the *Baylisascaris schroederi* -*GAPDH* (*Bs-GAPDH*) gene. Primers were designed according to the transcriptome data of *B. schroederi* (unpublished data sources [25]): BsGAPDH-F 5′-CGCGGATCCATGCTTTTAACAGCCGGTCACT-3′ and BsGAPDH-R 5′-CCGCTCGAGTCAGTGTTTGCTGATGTAAGCGA-3′, and BsTpx-F 5′-CGCGGATCCATGTCAAAGGCAGTGATTGGTAA-3′ and BsTpx-R 5′-CCGCTCGAGTCAATGTTTCTGGAAATAGGCTT-3′. The two pairs of primers both incorporated *Xho*I and *Bam*HI (TaKaRa, Dalian, China) restriction sites (underlined). After separation by gel electrophoresis and purification using a DNA purification kit (Novagen, Germany), amplified products were cloned into vector pMD-19T following the manufacturer’s protocol (TaKaRa, Dalian, China), transformed into *Escherichia coli* DH5α (Tiangen, Beijing, China) and subsequently sequenced (Invitrogen, Shanghai, China).

### 2.5. Sequence and Bioinformatic Analysis

Open Reading Frame (ORF) finder (www.ncbi.nlm.nih.gov/projects/gorf/) was applied to the cDNA sequences. ProtParam tools (http://web.expasy.org/protparam/) were used to predict the protein molecular weight, isoelectric point and solubility. Amino acid sequence alignment was performed using Clustal X software version 1.83 [26] and phylogenetic trees were constructed by the neighbor-joining method with MEGA software (version 5.0) [27]. Secondary structure was predicted by the SOPMA secondary structure prediction method (https://npsa-prabi.ibcp.fr/cgi-bin/npsa_automat.pl?page=npsa_sopma.html). Domains, motifs and active sites were analyzed using Predictprotein (https://www.predictprotein.org/).

### 2.6. Expression and Purification of Baylisascaris schroederi Thioredoxin Peroxidase and Glyceraldehyde-3-Phosphate Dehydrogenase

Recombinant proteins were expressed and purified as previously described [28]. Briefly, the PCR products were digested with *Bam*HI and *Xho*I (TakaRa) and ligated into the expression vector pET32a(+) (Novagen, Madison, WI, USA). Then, the plasmid was transformed into *E. coli* BL21 (DE3) and grown at 37 °C in Luria-Bertani medium containing ampicillin (100 μg/mL) until the OD_600 nm_ reached 0.6. Next, *E. coli* cells were induced with 1.0 mM isopropyl β-D-1-thiogalactopyranoside (IPTG) for 5 h at 37 °C. The cells were collected by centrifugation (4600× *g*, 10 min) and resuspended in lysis buffer [50 mM NaH2PO4 (pH 8.0), 10 mM Tris-HCl (pH 8.0), 100 mM NaCl]. The samples were then sonicated until they were no longer viscous. The recombinant proteins were purified using Ni-NTA resin as previously described, and the purity of protein was estimated by sodium dodecyl sulfate polyacrylamide gel electrophoresis (SDS-PAGE) [29].

### 2.7. Preparation of Polyclonal Antibodies

To obtain rabbit sera against recombinant Bs-GAPDH (rBs-GAPDH) and recombinant Bs-Tpx (rBs-Tpx) for western blotting and immunolocalization, four male New Zealand White rabbits were immunized with 50 μg of each of purified recombinant protein mixed with Freund’s complete adjuvant (Sigma, St. Louis, MO, USA), followed by two booster immunizations (2 weeks apart) using the same route and dose in the same adjuvant. Rabbit sera were collected 2 weeks after the final administration and stored at −20 °C. The immune sera against either rBs-GAPDH or rBs-Tpx was purified using HiTrap Protein A affinity chromatography (Bio-Rad, Hercules, CA, USA) and the specific IgG antibodies were obtained and preserved at −80 °C until use. 

### 2.8. Western Blot Analysis and Immunolocalization

Recombinant proteins and protein extracts of *B. schroederi* were separated by 12% SDS-PAGE and then transferred onto nitrocellulose membranes. The following immunoblotting steps were carried out as described elsewhere [30]. In brief, these two recombinant proteins were incubated with *B. schroederi*-positive panda sera and rabbit anti-recombinant protein immunoglobulin G (IgG), while the worm protein extracts were incubated with rabbit anti-recombinant protein IgG with a dilution of 1:100 (*v*/*v*) in 5% skim milk to test the antigenicity of proteins. Pre-immune rabbit serum and *B. schroederi*-negative panda serum were also included as negative controls.

For immunolocalization in *B. schroederi*, sections were preprocessed and the antiserum against fusion proteins was collected from rabbit using standard procedures [31]. Procedures were carried out as described elsewhere [32], with modified rabbit anti-recombinant antibodies (1:100) and fluorescein isothiocyanate (FITC)-conjugated goat anti-rabbit IgG (1:3000; Boster). Pre-immune rabbit IgG were applied for negative controls. After rinsing with PBS containing 0.05% Tween-20 (PBST), the stained samples were detected under a fluorescence microscope.

### 2.9. Development of Indirect ELISA

The optimum serum dilution and coated antigen concentration were determined through standard checkerboard titration procedures [33]. In brief, ELISA plates were coated with 100 μL/well of twofold diluted recombinant protein (dilution ranging from 1:50 to 1:6400) in carbonate buffer (0.1 M, pH 9.6) at 4 °C overnight. Then, all wells were blocked with 100 μL of 5% skim milk for 1 h at 37 °C. The serum of pandas infected with *B. schroederi* and negative serum, both at a dilution of 1:20 to 1:640 in PBS, were added to wells (100 μL/well) and incubated for 1 h at 37 °C. After rinsing with PBST (PBS + 0.05% Tween 20), HRP-labeled rabbit anti-panda IgG, diluted to 1:4000 with PBS, was used in the subsequent step. After rinsing again five times, 100 μL tetramethylbenzidine substrate was added into every well and incubated in the dark for 15 min. Finally, 2 M H_2_SO_4_ was applied to terminate the reaction and the absorbance was measured at 450 nm in a microplate reader. We chose the optimal working conditions which gave the highest P/N value [34]. In these conditions, the cut-off value of the indirect ELISA was calculated as the mean + three standard deviations using data from 24 negative serum samples from pandas.

### 2.10. Antibody Titer Detection of Mouse Serum

The optical density values of mouse sera (positive sera, *n* = 10; negative sera, *n* = 10) were measured with the established indirect ELISA, and used to calculate the P/N value for mouse serum. Positive serum was produced as follows: each mouse was given 1000 embryonated *B. schroederi* eggs by oral administration. Then, all mice were sacrificed and mouse anti-*B. schroederi* serum was collected for serodiagnostic assays 3 weeks after infection. 

### 2.11. Repeatability and Reproducibility of the Indirect ELISA

ELISA was carried out simultaneously with five positive serum samples to evaluate the repeatability (intra-assay variability) and performed continuously to detect the reproducibility (inter-assay variability). Each sample was repeated three times and subsequently the coefficients of variation (CV) were calculated.

### 2.12. Sensitivity and Specificity Analysis of the ELISA

The percentage sensitivity of this ELISA method was determined as positive×100/true positive, while the percentage specificity of the method was evaluated as negative×100/true negative.

### 2.13. Statistical Analysis

All data are presented as the mean ± standard deviation (SD). Statistical analyses were performed by *t*-test and Mann-Whitney *U* test using SPSS Statistics 20 (SPSS Inc., Chicago, IL, USA). *p*-values < 0.05 were considered significant. 

## 3. Results

### 3.1. Molecular and Characterization of Baylisascaris schroederi Thioredoxin Peroxidase and Glyceraldehyde-3-Phosphate Dehydrogenase

**Figure 1:**
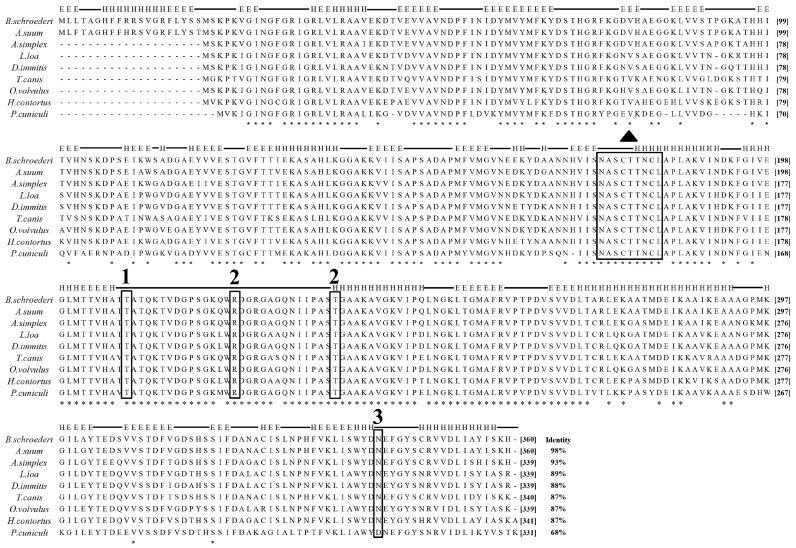
Multiple sequence alignment of *B. schroederi* GAPDH with homologues from other species. *Ascaris suum* GAPDH (ERG79426.1), *Anisakis simplex* GAPDH (AIT71762.1), *Loa loa* GAPDH (XP_020305933.1), *Dirofilaria immitis* GAPDH (AFL46382.1), *Toxocara canis* GAPDH (KHN89029.1)*, Onchocerca volvulus* GAPDH (AAB52599.1), *Haemonchus contortus*GAPDH (ADI46817.1), *Psoroptes cuniculi* GAPDH (unpublished). Amino acid residues in marked boxes are (1) GAP phosphate binding site, (2) inorganic phosphate binding sites, (3) NAD+ binding site, whereas (▲) indicates enzymatic binding site.

**Figure 2:**
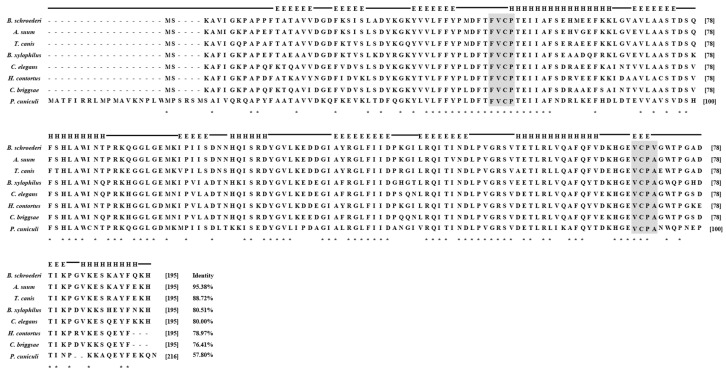
Multiple sequence alignment of *B. schroederi* thioredoxin peroxidase (BsTpx). *A. suum* (Q9NL98.1), *T. canis* (KHN73050.1), *Bursaphelenchus xylophilus* (ABW81468.1), *C. elegans Caenorhabditis elegans* (NP_872052.1), *H. contortus* (AAT28331.1), *Caenorhabditis briggsae* (XP_002630699.1), *Psoroptes cuniculi* (unpublished). The catalytic sites (FVCP-VCPA) of Tpx are indicated in light gray shading.

The entire ORF of the Bs-Tpx cDNA sequence was 588 bp, which encoded a 21.6 kDa protein. The ORF of Bs-GAPDH was 1083 bp and encoded a predicted 38.6-kDa protein. Multiple sequence alignments showed that Bs-GAPDH shared the highest amino acid similarity (98%) with GAPDH from *Ascaris suum* (GenBank: ERG79426.1) while the protein sequence of Bs-Tpx had 95% identity to the Tpx of *A. suum* (GenBank: AB058666.1). Moreover, multiple sequence alignment of GAPDH from *B. schroederi* showed that the identified substrate binding sites and conserved regions were consistent with those of homologues from other parasites (Figure 1); likewise, Tpx from *B. schroederi* contained a typical catalytic site (FVCP-VCPA) which was also found in homologues of other species (Figure 2). 

### 3.2. Expression and Identification of the Recombinant Proteins

**Figure 3:**
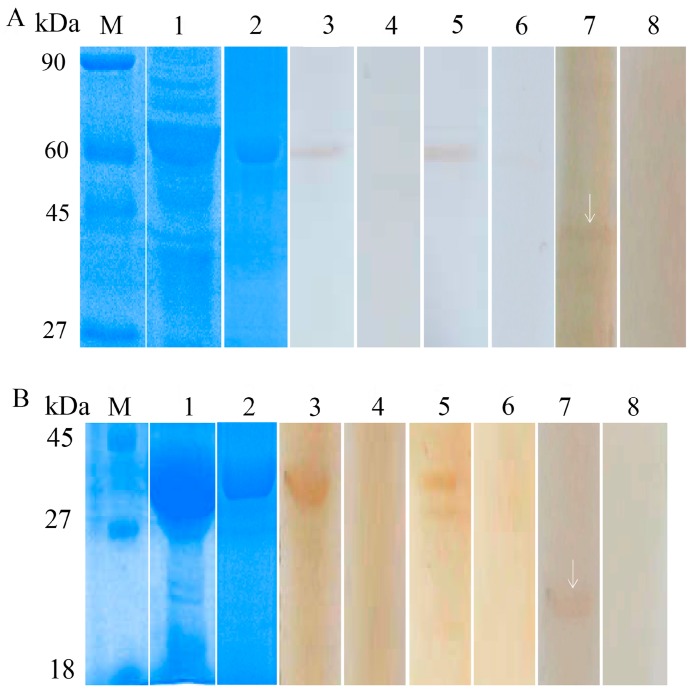
Sodium dodecyl sulfate polyacrylamide gel electrophoresis (SDS-PAGE) and western blot analysis of *B. schroederi* GAPDH (*Bs-GAPDH*) and Tpx (*Bs-Tpx*). (**A**) M: protein molecular weight markers; Lane 1: expression of recombinant *Bs-GAPDH* (r*Bs-GAPDH*) in *Escherichia coli*; Lane 2: purified rBs-GAPDH (4 μg); Lane 3–4: purified rBs-GAPDH (4 μg) incubated with anti-rBs-GAPDH rabbit IgG or pre-immune rabbit sera; Lane 5–6: purified rBs-GAPDH (4 μg) incubated with *B. schroederi*-positive panda sera or negative panda sera; Lane 7–8: extracts of *B. schroederi* (20 μg) incubated with anti-rBs-GAPDH rabbit IgG or pre-immune rabbit sera. (**B**) M: protein molecular weight markers; Lane 1: expression of recombinant *Bs-Tpx*(r*Bs-Tpx*) in *E. coli*; Lane 2: purified r*Bs-Tpx* (6 μg); Lane 3–4: purified r*Bs-Tpx* incubated with rabbit anti-rBs-Tpx IgG or pre-immune rabbit sera; Lane 5–6: purified r*Bs-Tpx* (6 μg) incubated with *B. schroederi*-positive panda serum or negative panda serum; Lane 7–8: extracts of *B. schroederi* (20 μg) incubated with rabbit anti-rBs-Tpx IgG or pre-immune rabbit sera. The arrows indicated the native non-fusion protein.

Recombinant *Bs*-GAPDH was expressed as a soluble protein with a molecular weight of approximately 57 kDa (Figure 3A, lane 1). Recombinant *Bs*-Tpx was expressed in inclusion bodies with a molecular weight of approximately 39 kDa (Figure 3B, lane 1). The constructs both contained a 17 kDa epitope tag fusion peptide, thus, the molecular masses of *Bs*-GAPDH and *Bs*-Tpx were ~40 kDa and ~22 kDa, respectively, similar to the masses predicted from their amino acid sequences. 

Western blot analysis showed that Bs-GAPDH (Figure 3A) and Bs-Tpx (Figure 3B) reacted with the sera of pandas infected with *B. schroederi.* Positive bands of 39 kDa and 57 kDa appeared when the purified rBs-Tpx and rBs-GAPDH were respectively incubated with rabbit anti-recombinant sera or *B. schroederi*-positive panda sera, manifesting an intense reactivity of these two fusion proteins. No band was observed when recombinant proteins were probed with negative panda sera or pre-immune rabbit sera. Additionally, the total worm protein extracted from *B. schroederi* was probed with anti-rBsGAPDH and anti-rBsTpx rabbit sera and the targeted bands of about 39 kDa (Figure 3A, lane 7) and 22 kDa (Figure 3B, lane 7) were also observed. No reactions were detected with the sera of naïve rabbit.

### 3.3. Immunolocalization of Endogenous Baylisascaris schroederi Thioredoxin Peroxidase and Glyceraldehyde-3-Phosphate Dehydrogenase

**Figure 4:**
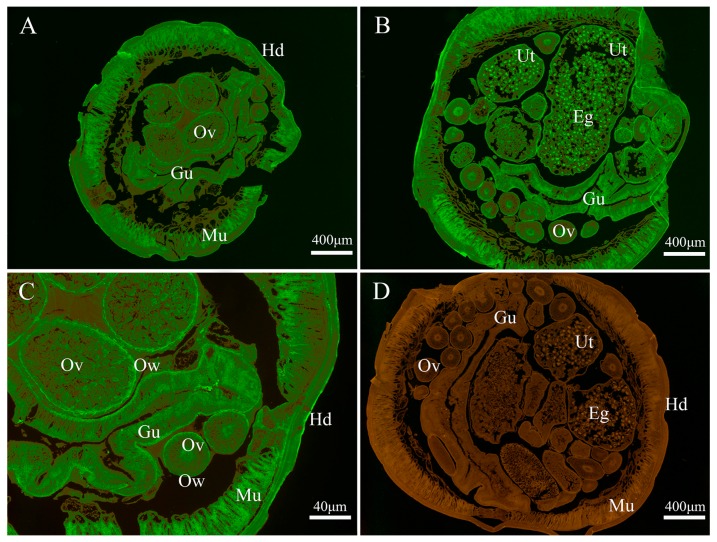
Immunolocalization of *B. schroederi* GAPDH *(Bs*-GAPDH). The sections (5-μm thickness) were incubated with rabbit anti-recombinant *Bs*-GAPDH (anti-r*Bs*-GAPDH) IgG (1:100; panels **A**–**C**), pre-immune rabbit IgG (1:100; panel **D**). Panel (**C**) represented the magnified part of panel (**A**). The endogenous *Bs*-GAPDH becomes visible with fluorescein isothiocyanate (FITC)-labeled goat anti-rabbit IgG (1:100). Abbreviations: Gu, gut; Mu, muscle; Ov, ovary; Hd, hypodermis; Ut, uterus; Eg, egg; Ow, ovarian wall.

**Figure 5:**
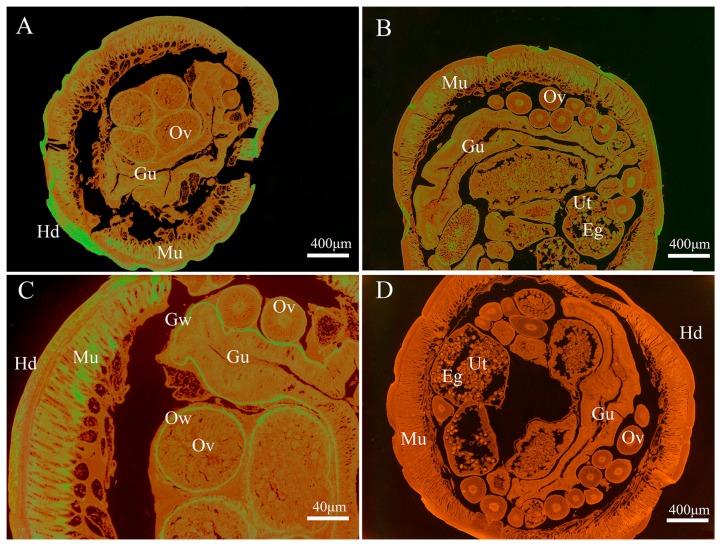
Immunolocalization of *B. schroederi* Tpx (Bs-Tpx). The sections (5-μm thickness) were incubated with rabbit anti-recombinant *Bs*-Tpx (anti-r*Bs*-Tpx) IgG (1:100; panels **A**–**C**), pre-immune rabbit IgG at (1:100; panel **D**). Panel (**C**) represented the magnified part of panel (**A**). The endogenous *Bs*-Tpx becomes visible with fluorescein isothiocyanate (FITC)-labeled goat anti-rabbit IgG (1:100). Abbreviations: Eg, egg; Ut, uterus; Mu, muscle; Hd, hypodermis; Ov, ovary; Ow, ovarian wall; Gu, gut; Gw, gut wall.

As shown in Figure 4, the endogenous Bs-GAPDH was found to be widely distributed in various tissues, particular in the gut, muscle, eggs, uterus wall and ovary wall (Figure 4). For the endogenous Bs-Tpx, the strong signal was mainly localized in the muscle, eggs, gut wall, uterus wall and hypodermis (Figure 5).

### 3.4. Establishment of Indirect ELISA

Indirect ELISA was applied to detect the potential of Bs-Tpx and Bs-GAPDH as diagnostic antigens. Checkerboard titration tests demonstrated that the highest P/N value was 2.343 when rBs-GAPDH was used at 4.4 μg/well and the serum dilution was 1:160. For rBs-Tpx, 1.065 μg/well of antigen and 1:20 dilution of serum were considered to be optimal, giving a P/N value of 3.488. In the optimized conditions, 24 negative panda serum samples were used to calculate the threshold value of these indirect ELISAs. All samples were measured in triplicate. The cut-off value of the rBsTpx-based ELISA was 0.161 (mean = 0.131, SD = 0.0101), and the cut-off value of the rBsGAPDH-based ELISA was 0.179 (mean = 0.135, SD = 0.0147).

### 3.5. Detection of the Antibody Titers of Mice Infected with Baylisascaris schroederi 

We used 10 mice artificially infected with *B. schroederi* to detect the antibody titers with the indirect ELISA. The P/N values of the mouse sera were between 5.061 and 9.357 when rBs-GAPDH was the coating antigen, while the P/N values were between 5.308 and 9.898 with rBs-Tpx.

### 3.6. Repeatability and Reproducibility of the Indirect ELISA

The rBs-GAPDH-based ELISA showed inter-assay and intra-assay variance coefficients ranging from 0.43% to 5.49% and 2.97% to 6.48% respectively. The inter-assay and intra-assay variance coefficients ranged from 1.18% to 4.96% and 3.02% to 6.86% respectively when rBs-Tpx was used as the coating antigen.

### 3.7. Specificity and Sensitivity of the Indirect ELISA

**Figure 6:**
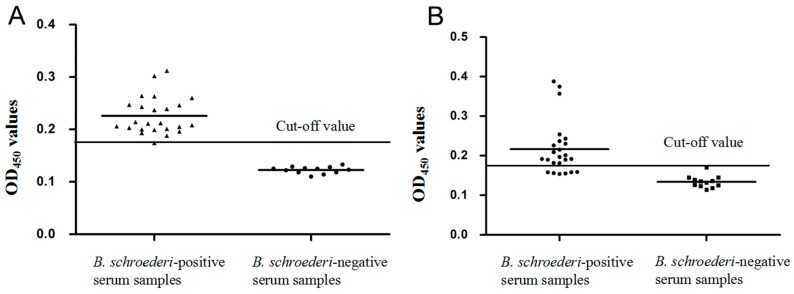
Indirect enzyme-linked immunosorbent assay (ELISA) for the detection of giant panda infected with *B. schroederi*. (**A**) Sensitivity and specificity of the indirect ELISA based on recombinant Bs-GAPDH. The cut off value is 0.179. Statistically significant differences between *B. schroederi*-positive sera and healthy giant panda sera were tested by *t*-test (*t* (34) = 26.016, *p* < 0.0001). (**B**) Sensitivity and specificity of the indirect ELISA based on recombinant Bs-Tpx. The cut off value is 0.161. Statistically significant differences between *B. schroederi*-positive sera and healthy giant panda sera (Mann–Whitney U, z = −4.632, *p* < 0.0001).

For rBs-GAPDH-based ELISA, 23 of 24 serum samples from pandas infected with *B. schroederi* were confirmed as positive and all naïve serum samples tested negative, based on the cut-off value of 0.179. Therefore, there was a sensitivity of 95.83% (23/24) and specificity of 100% for the rBsGAPDH-based ELISA (Figure 6A). A significant difference was observed between the positive group and the negative group (t(34) = 26.016, *p* < 0.0001). For the rBs-Tpx-based ELISA, 6 of 24 positive serum samples tested negative, and 1 of 12 naïve serum samples was determined as positive based on the cut-off value of 0.161, indicating that the sensitivity and specificity of the rBsTpx-based ELISA were 75.0% (18/24) and 91.7% (11/12) respectively (Figure 6B). A significant difference was observed between the positive group and the negative group (Mann–Whitney U, z = −4.632, *p* < 0.0001).

## 4. Discussion

The existing diagnostic methods for panda baylisascariasis, including sedimentation floatation and PCR detection, are based on detecting eggs separated from feces. There is a drawback of these methods as they cannot be used during the larval migration stage. No effective serological diagnosis method has been developed for panda baylisascariasis, especially in the early stage [35]. Tpx and GAPDH were confirmed to be associated with the immune responses of hosts against parasite infection and further have proved to have serodiagnostic potential in detection of *S. japonicum* and *Fasciola gigantica* [15,16,23]. Thus, our study cloned and expressed the *B. schroederi* Tpx and GAPDH proteins and tested their potential as novel serodiagnostic antigens against baylisascariasis in giant pandas. Western-blotting analysis showed that these two purified recombinant proteins could be recognized by corresponding anti-recombinant rabbit sera and positive sera from pandas infected with *B. schroederi*. Additionally, when the total protein extracts of *B. schroederi* were probed with anti-GAPDH and anti-Tpx rabbit sera, the targeted bands were clearly observed; notably, these two bands were smaller than the recombinant proteins because the latter containing a ~17 kDa epitope tag fusion peptide.

Immunofluorescence assay showed that GAPDH was widely distributed in *B. schroederi* tissue, especially in the gut, muscle and eggs. Often, the processes of growth and metabolism of parasites need a lot of energy provided by the glycolytic pathway. During the processes, the gut is key for *B. schroederi* to digest and absorb nutrition from the host. Hence, as a significant glycolytic enzyme, it is reasonable that GAPDH was widely distributed with the highest expression in the gut. Likewise, another key enzyme enolase from the nematode *Onchocerca volvulus* that functions in the glycolytic pathway was also mainly located in the gut [36]. Tpx was mainly distributed in uterus wall, hypodermis and eggs. As a species parasitizing the intestine of hosts, *B. schroederi* must resist oxidative stress, both from its own metabolism and the immune response of the host. Therefore, as a key enzyme in the antioxidant system, Bs-Tpx presents the contact area between *B. schroederi* and the host and often experiences the highest oxidative stress, it is reasonable that the expression of Tpx was highest in the gut wall, uterus wall and hypodermis. Similar results were found in a study of Tpx in *Echinococcus granulosus* [37].

In the optimum conditions, the indirect ELISA method based on Bs-GAPDH was better than that based on Bs-Tpx in terms of sensitivity and specificity. In nature, pandas are mainly infected with two species of parasites, *B. schroederi* and the mite *Chorioptes panda* [38]. Although there is no any sequence information on GAPDH and Tpx for *C. panda*, we found that the GAPDH and Tpx of the mite *Psoroptes cuniculi* only shares 68% and 57.8% identity with their homologs from *B. schroederi*. Therefore, in consideration of the difference between these two parasites and the facts that pandas are rarely infected solely with *C. panda* and that this study was a preliminarily assessment of the potential diagnostic value of the ELISA-based methods, cross-reactivity was not assessed in this work. 

The immune system of giant pandas might be different from those of other mammals. Montali et al. demonstrated that canine distemper vaccine may have a good immunogenicity for some species, but not for pandas [39]. The antibody titers of two pandas inoculated with canine distemper vaccine were both lower than 1:5. A similar phenomenon was that there was no significant increase of the neutralizing antibody of pandas inoculated with canine distemper vaccine; indeed the antibody titers were generally low and did not last long, and the antibody could not even be detected in half of the pandas [40]. In contrast, the neutralizing antibody titers of canines increased rapidly after inoculation, and the antibody titers of red panda and mouse could rapidly rise to 236.8 and 102,400 respectively [41,42]. These results suggest that low antibody titer may be normal in pandas. In this study, we found the similar phenomenon that the antibody titer of pandas infected with *B. schroederi* was low and we speculate that this might be the result of the different immune system in giant pandas. It was reported that an ELISA method based on a hemoprotein from *A. suum* had been developed for detecting ascariasis and the P/N value in that test was generally greater than that calculated in our study [43]. *B. schroederi* is a close relative of *A. suum*, but there was an obvious difference in the antibody titers of its hosts, which further evidenced that the low antibody titers of pandas may be species-specific. Thus, to verify the feasibility of our method, we also tested the antibody titers of 10 positive mouse serum samples as well as 10 naïve mice serum samples through the ELISAs established in this study. The P/N values for mice were all greater than those for pandas, indicating that the method is feasible. No explanation of the low antibody-titers in pandas is available, and this phenomenon requires further investigation.

## 5. Conclusions

The full-length cDNAs encoding the Bs-Tpx and Bs-GAPDH were identified and characterized and then recombinant proteins were obtained using a prokaryotic expression system. We found that these two proteins were widely distributed in *B. schroederi*, especially Bs-GAPDH. Meanwhile, Bs-GAPDH-based indirect ELISA was sensitive and specific for detection of baylisascariasis. Recombinant Bs-Tpx was an unsuitable diagnostic antigen. These results showed that Bs-GAPDH protein may be a potential diagnostic antigen for an ELISA-based method for the detection of *B. schroederi* in giant pandas.
